# Frequency of Uropathogens and Antimicrobial Susceptibility in Childhood Urinary Tract Infection at Kamenge University Hospital, Bujumbura, Burundi

**DOI:** 10.24248/EAHRJ-D-16-00331

**Published:** 2017-03-01

**Authors:** Joseph Nyandwi, Sébastien Manirakiza, Eugàne Ndirahisha, Jean Baptiste Ngomirakiza, Désiré Nisubire, Emmanuel Nduwayo, Hélàne Bukuru

**Affiliations:** a Département de Médecine Interne, Le Centre Hospitalo-Universitaire de Kamenge, Bujumbura, Burundi; b Unité d’Hémodialyse, Burundi Kidney Care Clinic, Bujumbura, Burundi; c Département d’Imagerie Médicale, Le Centre Hospitalo-Universitaire de Kamenge, Bujumbura, Burundi; d Département des Laboratoires, Le Centre Hospitalo-Universitaire de Kamenge, Bujumbura, Burundi; e Département de Pédiatrie, Le Centre Hospitalo-Universitaire de Kamenge, Bujumbura, Burundi

## Abstract

**Background:**

Increasing resistance to antimicrobials is a worldwide problem. The aim of our study was to determine the pathogens and antimicrobial susceptibility of bacteria causing urinary tract infection (UTI) in children.

**Methods:**

This is a prospective cohort study conducted over a 10-month period with 101 children hospitalised at Kamenge University Hospital for acute UTI. The infections were confirmed by Kass urinalysis criteria, and culture and susceptibility antibiotic tests were performed for isolated microbial agents.

**Results:**

Frequency of UTI in the overall population of children hospitalised at Kamange University Hospital was 8.4%. Of the 101 children with UTIs, 87 (86.1%) were under the age of 24 months. Diagnosis of pyelonephritis (82%) was the most common, followed by cystitis (18%). *Escherichia coli* (82%) was the most frequent pathogen causing UTI. We found *E coli* and *Klebsiella pneumonia* to be resistant to aminopenicillins (100%), cotrimoxazole (98.2%, 100%), Augmentin (amoxicillin/clavulanic acid) (70.5%, 80%), cefotaxime (45.8%, 28.6%), cefuroxime (36.8 to 45.5%, 50%), fluoroquinolones (33.3 to 53.6%, 28.6 to 50%), gentamicin (27.5%, 20%), and nitrofurantoin (9.3%, 50%).

**Conclusion:**

*E coli* is the main causal agent of UTI in childhood with a high resistance to antibiotics. Appropriate antibiotics for empiric therapy should be based on local circulating bacterial strains and resistance profiles.

## INTRODUCTION

Antimicrobial resistance to common bacterial infections has become an increasingly challenging worldwide problem, as many currently available drugs have been found to have little or no effect on new resistant bacteria.^1^ Unfortunately, in many regions of the world, this issue is compounded by inadequate surveil-lance methods and a lack of accurate and reliable data, particularly in sub-Saharan Africa.^1^ Information concerning the antimicrobial susceptibility for bacteria causing urinary tract infections (UTIs) in children is more limited. UTI is one of the most common infection in children, and as many as 2% of children are diagnosed with at least 1 episode by the age of 10 years.^2^ Current research shows a range of frequencies of UTI infection in children: Adonis-Koffy et al^3^ reported the frequency of 0.77% in Abidjan, Côte d’Ivoire; Bourskraoui et al^4^ noted the frequency of 1.33% in Marrakech, Morocco; and Taques et al^5^ found a high prevalence between 5 and 10% in France. The prevalence of UTI in children is variable, based largely on sex and age. Within the first 12 months of life, the risk of UTI has been estimated at 6% of girls and 3% of boys. Between 12 and 24 months, the difference between the sexes increases with incidence estimated at 8% of girls and 2% of boys.^2^ Each year, 150 million UTIs are reported worldwide with estimated cost of more than 6 billion US dollars.^6,7^ Clinical diagnosis of UTI is not easy during childhood. The signs and symptoms in infants and young children are variable and nonspecific of urinary tract, which can delay diagnosis and treatment.^8^ Empirical and appropriate antimicrobial therapy should lead to rapid recovery and avoidance of complications. Delayed or incorrect antibiotic treatment may result in recurrence, particularly in children with renal abnormalities who risk developing progressive renal damage with hypertension and chronic renal failure, which can affect growth and quality of life of the child in long run.^9,10^ The antibiotic choice for empiric therapy should be based on local circulating bacterial strains and resistance profiles. In Burundi, antibiotic resistance has become a growing concern, particularly in children. Our research focused on the pathogens and antimicrobial susceptibility of bacteria causing UTIs in children hospitalised in paediatric service at Kamenge University Hospital, Bujumbura, Burundi.

## METHODS

### Design and Setting

We conducted a prospective cohort study of children hospitalised for UTIs with a positive urine culture in the paediatric service at Kamenge University Hospital from 1 January to 1 November 2013. A UTI was defined by the Kass criteria as leukocyturia ≥ 10^4^ cells/ml and bacteriuria ≥ 10^5^ cells/ml with the presence of a single bacterial pathogen.^11^

### Study Population

The study population began with the 1,200 children who had been hospitalised in the paediatric service at Kamenge University Hospital during the study period. Children aged 0 to 16 years with signs and symptoms leading to suspect acute UTI—including temperature ≥ 38°C, chills, frequency of urination, dysuria, urgency to urinate, suprapubic and/or flank tenderness, and fever with unknown source—with clinical evidence of sepsis were included in the study. We excluded children who had symptoms or signs implicating another infection source in order to rule out asymptomatic bacteriuria accompanied with other infections. Patients who had been on antibiotics for at least 3 days were also excluded. A total of 156 children provided samples for urinalysis. UTIs were confirmed for 101 children corresponding to our sample.

### Urine Collection and Transport

Urine samples were collected from younger children and infants using sterile urine bags, and from children older than 24 months using midstream clean-catch method. For the younger population, urine bags were initially placed by trained nurses, using standard perineal cleansing procedures. Parents were trained how to place another, according to the same cleansing procedures, if the urine bags were rejected or separated from the skin, which could result in leakage or stool contamination. Midstream clean-catch specimens were obtained by trained parents using a sterile urine container, taking care that the perineum did not touch the inside of the container. Within 30 minutes of collection, specimens were sent in ice bags for analysis to the laboratory of Kamenge University Hospital. Specimens were processed promptly or refrigerated at 4°C for a maximum of 12 hours before being analysed.

### Laboratory Methods

Microscopic and macroscopic tests assessed the color and turbidity of urine and the number of leukocytes and flora on Malassez cell, respectively. Samples containing ≥10 ^4^/ml of leukocytes were cultured on a selective medium Eosin Methylene Blue (EMB) Agar (Titan Media, India) and on a non-selective medium cysteine lactose electrolyte deficient (CLED) (Titan Media, India) with an incubation period of 18 to 24 hours at 37°C. Cultures of a single organism with a count of ≥10 ^5^ colony-forming units/ml were considered to represent infection and were then identified by Gram-stain to distinguish the bacilli from the cocci. The biochemical characteristics of Gram-positive bacilli were determined on a gallery including Kligler Iron Agar (glucose, H_2_S, lactose, gas) (Titan Media, India), Mannitol mobility test medium, Simmons Citrate Agar (Fortress Diagnostics, UK) and Urea Indole Medium (HiMedia Laboratories, India). The characteristics of Gram-positive cocci were determined by culture on mannitol salt agar, or Chapman medium, and D coccossel agar. Routine diagnostic antimicrobial susceptibility results were determined using the agar disk-diffusion method, in accordance with Clinical and Laboratory Standards Institute (CLSI) recommendations.^12^ Colonies from EMB were inoculated on sterile Mueller Hinton Agar (HiMedia Laboratories, India) using antibiotic discs and then incubated at 37°C for 18 to 24 hours. The antibiotics tested during this process were ceftazidime (30 μg), cefuroxime (30 μg), furadantine (300 μg), cefotaxime (30 μg), ciprofloxacin (10 μg), cloxacillin (4 μg), tetracycline (30 μg), erythromycin (15 μg), nalidixic acid (30 μg), Augmentin (amoxicillin 20 μg and clavulanic acid 10 μg), amoxicillin (25 μg), cotrimoxazole (1.25/3.75 μg), and gentamicin (10 μg). Following the incubation period, results were determined by measuring the diameter of the inhibition of zone. Zones of inhibition greater than 10 mm were considered sensitive, 5 to 10 mm moderately sensitive, and no zone of inhibition resistant.^13^

### Data Analysis

For this analysis, bacteria with intermediate resistant were not considered as resistant. Data were analysed using EpiInfo (Version 3.5.3). The proportions were compared using both Fisher's exact test and chi-square test to examine the difference in antimicrobial susceptibilities, a *P* value of <0.05 was used as a cut-off point for statistical significance.

### Ethics

The urine samples were taken only after researchers explained the purpose of the study to every parent of a child, and verbal and free consent was given.

## RESULTS

During the study period, 1,200 children were hospitalised in paediatric service at Kamenge University Hospital, of whom 700 (302 boys and 398 girls) were aged ≤ 24 months and 500 (240 boys and 260 girls) were aged more than 24 months. After applying the exclusion criteria and conducting a series of tests, 101 children (8.4%)—50 boys and 51 girls—diagnosed with UTIs were included in the study. The study population was comprised of 87 children (86.1%) aged ≤ 24 months—47 (46.5%) boys and 40 (13.9%) girls—and 14 children (13.9%) aged more than 24 months—3 (3%) boys and 11 (10.9%) girls (Table 1). A peak frequency of UTI was observed between the ages of 7 and 9 months, which gradually decreased and remained stable after the age of 30 months. The UTIs were defined by the site of predominant alteration, resulting in 83 cases of pyelonephritis (82%) and 18 cases of cystitis (18%).

**TABLE 1.** Sample Distribution by Age and Sex (N=101)

Sex	No. ≤ 24 Months	No. > 24 Months	Total
Male	47	3	50
Female	40	11	51
Total	87	14	101

### Organisms

We noted that 96 (95%) of isolates were enterobacteriaceae, of which 82% were *E coli*, 10% were *Klebsiella pneumoniae*, and 3% were *Proteus mirabilis*. Other bacteria isolated were *Staphylococcus aureus* (3%), *Enterococcus faecalis* (1%), and *Pseudomonas aeruginosa* (1%) (Table 2).

**TABLE 2.** Frequency of Bacteria Responsible for Urinary Tract Infections

Bacteria	Frequency	Percent
*Escherichia coli*	83	82
*Klebsiella pneumoniae*	10	10
*Proteus mirabilis*	3	3
*Staphylococcus aureus*	3	3
*Enterococcus faecalis*	1	1
*Pseudomonas aeruginosa*	1	1
**Total**	101	100

### Antimicrobial Susceptibility Patterns

The antimicrobial susceptibilities of the most common organisms, *E coli* and *K pneumoniae*, are shown in Table 3. *E coli* isolates were resistant to cotrimoxazole in 56 of 57 (98.2%) of cases followed by Augmentin in 55 of 78 (70.5%), cefotaxime in 27 of 59 (45.8%), cefuroxime in 20 of 44 (45.5%), ciprofloxacin in 25 of 75 (33.3%), gentamicin in 14 of 51 (27.5%), and nitrofurantoin in 4 of 43 (9.3%). *K pneumoniae* isolates presented high resistant to cotrimoxazole in 10 of 10 (100%) cases followed by Augmentin in 8 of 10 (80%), cefuroxime in 2 of 4 (50%), ciprofloxacin in 2 of 6 (33.3%), cefotaxime in 2 of 7 (28.6%), gentamicin in 1 of 5 (20%), and nitrofurantoin in 1 of 2 (50%). For all antibiotics tested, no observed statically significant differences were found in resistance prevalence between *E coli* and *K pneumoniae* (Table 3).

**TABLE 3.** Antibiotic Resistance of *Escherichia Coli* and *Klebsiella Pneumoniae*

	*E Coli*		*K Pneumoniae*		
Tested Drugs	No. of Tests Made	Resistance No. (%)	No. of Tests Made	Resistance No. (%)	*P* Value
Augmentin	78	55 (70.5)	10	8 (80)	.80
Cotrimoxazole	57	56 (98.2)	10	10 (100)	1.00
Cefuroxime	44	20 (45.5)	4	2 (50)	1.00
Cefotaxime	59	27 (45.8)	7	2 (28.6)	.71
Ciprofloxacin	75	25 (33.3)	6	2 (33.3)	1.00
Gentamicin	51	14 (27.5)	5	1 (20)	1.00
**Nitrofurantoin**	43	4 (9.3)	2	1 (50)	.27

## DISCUSSION

This study has shown the prevalence of isolation and antibiotic resistance pattern of uropathogenic bacteria in paediatric service at Kamenge University Hospital. Our results were compared with those of other studies of UTIs in hospitalised children. According to our findings, frequency of UTI among hospitalised children at Kamenge University Hospital was 8.4%. In France, Taques et al^5^ also found a high prevalence, between 5 to 10%, among the children in their study. Interestingly, Adonis-Koffy et al^3^ reported the frequency of 0.8% in children care for in the paediatric service of the Centre Hospitalier et Universitaire de Yopougon in Abidjan, Côte d’Ivoire, and Bourskraoui et al^4^ noted the frequency of 1.3% in children cared for in the paediatric services of the Teaching Hospital Mohammed VI in Marrakech, Morocco. The low prevalence of the 2 African studies has been explained by faulty diagnosis, and is therefore probably underestimated.^3,4^ In contrast, the high prevalence of UTIs in our study can be attributed to easily accessible diagnostics and free care for children under 5.

According to our research, UTIs are more prevalent in boys before the age of 2 years and in girls after the age of 2 years. In our sample, of the 87 children (86.1%) aged ≤ 24 months, 47 (54%) were boys. Of the 14 remaining children older than 2 years, 11 (78.5%) were girls. These results are similar to those of previous studies. In the study of Ferjani et al, 80% of children were infants.^14^ In 2010, Bouskraoui et al found that infants accounted for 70.9% of UTIs of which 55% were boys; in children older than 2 years, UTIs in girls predominated preschool (68%) and school age (76%) populations.^4^ In 105 cases in Burundi, Maloba found that 60% were infants aged less than 18 months of which 66.2% were boys; of those who were aged 18 months or older, 40% were girls.^15^ The pre dominance of UTIs in male infants can be attributed to the increased incidence of malformative uropathies and vesicoureteral reflux during this timeframe. Before age of 2 years, the infant preputial sac is still not free of the glans, which can lead to a stagnation of urine around the meatus causing a favorable bed for the local growth of bacteria and inflammation that cause phimosis, if the emptying of the bladder is incomplete, contamination of male meatus is exaggerated by the preputial sac. However, with age the foreskin retracts more easily and exposure to infection is reduced. Accordingly, the risk of infection could be reduced up to 10 times by circumcision or by daily retraction of foreskin before 6 months.^16–18^ At preschool age, the higher female prevalence reflects potential hygiene challenges related to a urogenital tract with a short urethra and the location of the urethral and vaginal meatus so close to the anal meatus. In our study, the main cause of UTIs in children was enterobacteriaea (95%): *E coli* (82%), *K pneumoniae* (10%), and *P mirabilis* (3%). Other bacteria were found at a lesser level: *S aureus* (3%), *P aeruginosa* (1%), and *E faecalis* (1%). *E coli* was the most frequent bacteria isolated, as found in other studies.^3,4,14,15,18–21^ The predominance of *E coli* can be attributed to its ability to attach to the urinary tract endothelium.

Depending on the sensitivity of the tests, the 2 primary isolated bacteria—*E coli* and *K pneumoniae*—had variable rates of resistance to all tested antibiotics. For aminopenicillins (Augmentin, ampicillin, and amoxicillin), resistance varied from 70.5 to 100% for *E coli* and 80 to 100% for *K pneumoniae*. High resistance was also found by other authors: Adonis-Koffy et al showed the *E coli* resistance ranged from 68% to 100%,^3^ Bourskroui et al demonstrated a range of 58 to 68%,^4^ and Ferjani et al discovered a range of 65 to 78%.^14^ In 2002, researchers at Kamenge University Hospital found the antibiotic resistance of *E coli* was 50% for ampicillin and 47% for Augmentin.^15^ Furthermore, the klebsielles are naturally resistant to aminopenicillin. Due to these high levels of resistance, aminopenicillins are no longer the drug of choice for the treatment of UTIs. Cotrimoxazole resistance was also high in our study—98.2% for *E coli* and 100% for *K pneumoniae*—while in a previous study at Kamenge University Hospital the range for all bacteria was between 82 and 100%.^15^ However, in certain African studies, cotrimoxazole was found more or less effective for certain serotypes of *E coli*.^4,14^ In contrast, second- and third-generation cephalosporins showed resistance to *E coli* (45%) and to *K pneumoniae* (28 to 50%), while other pathogens were 100% sensitive to these antibiotics. Our findings differ from studies where cephalosporins are still proven to have an efficacy ranging from 95% to 100%.^3,4,14^ It has been 10 years since there was no resistance to cephalosporins in Burundi.^15^ Similarly, in France, cephalosporin resistance is < 1%.^22^ The increasing resistance to cephalosporins in our study could be due to the emergence of new resistant strains related to incorrect prescribing, by association with other antibiotics; purchasing drugs without a prescription; or poor antibiotic adherence, by taking insufficient doses to address the infection. We found that gentamicin, an aminoglycoside regularly associated with other antibiotics for empiric therapy, was resistant to *E coli* (27.5%), *K pneumoniae* (20%), and *S aureus* (50%) at the lower levels, and at 100% for other bacteria. In other studies, gentamicin resistance was less than 10%.^3,4,14^ The rate of resistance to ciprofloxacin was 33.3% for *E coli*, 28.6% for *K pneumoniae*, and ranged from 33.3% to 100% for other bacteria, except *P aeruginosa* for which the sensitivity was 100%. Other reported findings show that quinolone resistance to uropathogenic organisms ranged from 0 to 10%.^3,4,14,15^ In our study, nitrofurans (nitrofurantoin or Furadentine) were efficient with less than 10% of resistance to *E coli*. Our observation supports previous studies indicating that nitrofurantoin exhibited less than 10% of resistance rates in the most countries investigated.^3,4,15,20,23–25^ This may have been due to the fact that this antibiotic has not been widely used for treating UTI cases. Therefore, nitrofurantoin may be a good option for the empirical treatment of UTI in Burundi. However, it should not be used to treat UTI in febrile infants because it is excreted in the urine and does not achieve therapeutic concentrations in the bloodstream.^26^

For more than 10 years, we have observed the progressive rates of antibiotic resistant in Burundi. In our study, the higher resistance rates to all antibiotics tested, with the exception of nitrofurantoin, may be explained by high and uncontrolled usage of these antimicrobial agents, since these antibiotics are also prescribed for other infections.

Methods for testing antimicrobial susceptibility and discovering novel antimicrobial agents have been extensively used. In our study, we used the agar disk-diffusion method often used in many clinical microbiology laboratories for routine antimicrobial susceptibility testing. However, this method cannot accurately test all fastidious bacteria, due to their complex nutritional requirements. Antibiograms are overall profiles of antimicrobial susceptibility testing results, which categorise bacteria as susceptible, intermediate, or resistant. It is a typing tool based on the resistance phenotype of the microbial strain tested. The test outcomes guide clinicians in the appropriate selection of initial empiric treatments. However, this method is limited in that it cannot distinguish between bactericidal and bacteriostatic effects and is not as appropriate to determine the minimum inhibitory concentration (MIC) as the antimicrobial gradient method (Etest). The latter method is used to determination the MIC of antibiotics, antifungals, and antimycobacterials,^27^ and is useful for quantitative determination of susceptibility of bacteria to antibacterial agents. The Etest can confirm or detect a specific resistance phenotype like extended spectrum beta-lactamases (ESBL) producers.^28^ This technique has shown a good correlation between the MIC values determined by Etest and those obtained by broth dilution or agar dilution methods.^29,30^ The Etest is simple to implement and can be performed to investigate the antimicrobial interaction between 2 drugs,^31^ which is why it is routinely requested by clinicians. However, if many drugs need to be tested, the Etest process becomes costly. In contrast, the agar disk-diffusion test offers simplicity, low cost, the ability to test a large number of microorganisms and antimicrobial agents, and the ease to interpret results provided, giving it many advantages over other methods. Due to the good correlation between the in vitro data and the in vivo evolution this method demonstrated the great interest in patients who suffer from bacterial infection of an antiobiotherapy based on the antibiogram of the causative agent.^32,33^

This study has limitations. Our study focused on a range of bacteria known for causing UTIs in children, validating *E coli* as the primary strain of concern. However, it would be important to further examine the subtypes of *E coli* to show which is resistant to available antibiotics. Likewise, additional research would be useful to identify ESBL producers since they indicate conferred resistance to antibiotics and are associated with poor outcomes.

## CONCLUSION

UTI is a common disorder in a paediatric hospitals. During the period studied, the prevalence of UTIs in children at Kamenge University Hospital was 8.4%. Enterobacteriaceae were determined to be the main uropathogenic bacteria, with *E coli* being the main causative agent in over 80% of cases. Since 2002, Kamenge University Hospital research has determined that bacterial agents have shown absolute resistance to widely prescribed antibiotics such as cotrimoxazole and aminopenicillins, and increasing resistance to cephalosporins, aminoglycosides, and most fluoroquinolones, except nitrofurantoin. This work highlights the critical importance of pathogen and resistance surveillance over time.
